# The knitting community-based trial for older women with osteoarthritis of the hands: design and rationale of a randomized controlled trial

**DOI:** 10.1186/s12891-018-1965-2

**Published:** 2018-02-14

**Authors:** Paulette Guitard, Lucie Brosseau, George A. Wells, Nicole Paquet, Gail Paterson, Karine Toupin-April, Sabrina Cavallo, Sibel Z. Aydin, Guillaume Léonard, Gino De Angelis

**Affiliations:** 1 School of Rehabilitation Sciences, Faculty of Health Sciences, University of Ottawa, 451 Smyth Road, Ottawa, ON K1H 8M5 Canada; 2 School of Epidemiology, Public Health and Preventive Medicine, University of Ottawa, Ottawa, ON Canada; 3 The Arthritis Society, Ottawa Office, Ontario Division, Ottawa, ON Canada; 4 Children’s Hospital of Eastern Ontario Research Institute, Department of Pediatrics, Faculty of Medicine and School of Rehabilitation Sciences, Faculty of Health Sciences, University of Ottawa, Ottawa, ON Canada; 5 School of Rehabilitation Sciences, University of Ottawa, Ottawa, ON Canada; 6 Ottawa Hospital, Ottawa, ON Canada; 7 Val-des-Monts, University of Sherbrooke; and researcher, Research Center on Aging, Sherbrooke, QC Canada

## Abstract

**Background:**

The prevalence of hand osteoarthritis (HOA) has been reported to be higher amongst women over 50 years old (66%) compared to men of the same age (34%). Although exercise therapy has been shown effective in reducing symptoms and disability associated with HOA, adherence to treatment programs remains low. The primary objective of this RCT is to examine the effectiveness of a 12-week knitting program for morning stiffness (primary outcome) and pain relief (secondary outcome) 2 h post-wakening in females (aged 50 to 85 years old) with mild to moderate hand osteoarthritis (HOA).

**Methods/design:**

A single-blind, two-arm randomized controlled trial (RCT) with a parallel group design will be used to reach this objective and compare results to a control group receiving an educational pamphlet on osteoarththritis (OA) designed by the Arthritis Society. The premise behind the knitting program is to use a meaningful occupation as the main component of an exercise program. The knitting program will include two components: 1) bi-weekly 20-min knitting sessions at a senior’s club and 2) 20-min home daily knitting sessions for the five remaining weekdays. Participants assigned to the control group will be encouraged to read the educational pamphlet and continue with usual routine. Pain, morning stiffness, hand function, self-efficacy and quality of life will be measured at baseline, six weeks, 12 weeks (end of program) with standardized tools. We hypothesize that participants in the knitting program will have significant improvements in all clinical outcomes compared to the control group.

A published case study as well as the preliminary results of a feasibility study as examined through a 6-week pre-post study (*n* = 5 women with HOA) involving 20-min daily knitting morning sessions led to this proposed randomized controlled trial research protocol. This article describes the intervention, the empirical evidence to support it.

**Discussion:**

This knitting RCT has the potential to enhance our understanding of the daily HOA symptoms control and exercise adherence, refine functional exercise recommendations in this prevalent disease, and reduce the burden of disability in older women.

**Trial Registration:**

(ACTRN12617000843358) registered on 7/06/2017.

## Background

Osteoarthritis affects a large proportion of the world population and hand osteoarthritis (HOA) represents the highest age-standardized total prevalence (43.3%) in terms of joint site compared to hip OA (23.9%) and knee OA (10.9%) [1]. A higher age-standardized total prevalence of HOA is observed amongst women over 50 years old (65.8%) compared to men of the same age (34.2%) [2]. The prevalence of HOA is expected to increase with the aging of the population [3]. HOA is recognized as a significant cause of disability resulting in activity and participation limitations as well as reduced quality of life (QoL) [4, 5] due to presence of pain, morning stiffness, tenderness and swelling of the fingers joints, diminished grip strength and psychological problems [2, 6].

Active hand exercises (i.e. strengthening exercises and yoga) have been shown to be an effective strategy to reduce morning stiffness [7, 8] and pain [9–11], increase range of motion [12, 13] grip and/or pinch strength [7–9, 11–16] improve hand functional status in HOA [8, 10] and reduce disease activity [7]. To our knowledge, no studies examined the effect of functional activities in the management of HOA. Several authors suggested that there is an urgent need for more trials of nonpharmacological and nonsurgical interventions for HOA [8, 17–20].

Isometric contractions (i.e. a type of muscle contraction involved knitting) have been shown effective to reduce pain perception, both in healthy individuals and in patients suffering from chronic pain [21, 22]. For healthy individuals, long duration activity (30 min) seems to be necessary to trigger exercise-induced hypoalgesia [22, 23]. For patients with chronic pain, meaningful change in pain is observed both for high and low intensity exercises protocols [21, 24, 25]. Psychosocial aspects can also contribute to the beneficial effects of exercise on pain [22, 26, 27].

According to the Canadian Model of Occupational Performance and Engagement (CMOP-E), [28] occupations bring meaning to life. Occupational therapy explores the therapeutic potential of occupations; it is believed that the power and the positive effects of occupations are greater when client can choose, control and get a sense of accomplishment through them. The movements typically incorporated in hand therapy for HOA (finger exercises [9] and muscle activation [22, 23] are also present in knitting. Knitting can be considered a purposeful and meaningful occupation. As such, knitting may be more appealing and meaningful for elderly women to participate in compared to regimented exercises programs. This study embeds the functional activity of knitting in an exercise program that may demonstrate greater adherence than regimented hand exercises [29]. Knitting also represents a promising activity for older individuals suffering from HOA pain via its potential effect on psychological and social factors (e.g. positive effect on mood and social isolation, particularly if knitting is done at a senior’s club). Indeed, it could be more appealing, motivating, enjoyable and rewarding to perform an activity that is highly appreciated [30] than a series of prescribed therapeutic exercise. To our knowledge, no studies have examined the effect of functional activities in the management of HOA.

In this research protocol, knitting is used in a larger context. It is prescribed and is structured in terms of frequency and duration, but not regarding intensity, style of knitting or needles holding (respect of personal fashion and willingness)) and is a replacement of a traditional prescribed exercise program during a therapy. A therapeutic aim would be to prevent morning stiffness as much as possible to maintain a regular level of daily activities. The proposed randomized controlled trial (RCT) will incorporate meaningful occupation (i.e. knitting) within a structured and supervised context.

The results of a recent case study (Brosseau & Leonard, 2017) revealed that a 12-week low intensity knitting program is a promising self-management strategy for mild to moderate HOA. Performing daily early morning knitting over 12 weeks resulted in a 50% short-term improvement in daily pain and joint stiffness relief in bilateral osteoarthritic fingers of an 86-year old woman who had been living with the disease for at least 40 years. She showed strong adherence to the program by participating in all knitting sessions (100%). This individual reported improved patient global assessment [31], strong goal attainment [32], and improved self-efficacy in managing her arthritis pain (60% improvement) [33].

Following the results of this case study, a feasibility study was conducted. The preliminary results showed also that the five elderly women (80 to 87 years old) had a 100% adherence rate during a 6 weeks knitting intervention. The six-week knitting intervention, the selected measurements and the adapted logbook were deemed useful, easy to follow and were well-accepted by older women with HOA. Furthermore, the five participants reported an average daily immediate relief of 45% and 77% respectfully for pain and morning stiffness (i.e. great effect on morning stiffness). These daily improvements were maintained over two as well as four consecutive hours. Four of five participants loved to knit and had knitted in the past, but had not knitted for at least two months. The fifth participant never knitted in her life. She did not enjoy it, but nevertheless adhered to the program until the completion of the study.

The proposed knitting program is based on: 1) evidence stemming from physiological studies [34–36]; 2) recommendations for individuals over 50 years old with OA [37] and specifically with HOA [12]; 3) evidence-based general exercise protocols for HOA [5, 38, 39] and OA of the thumb [40–42]; 4) current RCTs on therapeutic exercises for HOA [9, 14] and on the case study parameters described above [43]. In the proposed study, knitting will be a low-intensity hand exercise therapy comprising bilaterally dynamic and isometric movement of fingers, thumbs and wrists. Despite knitting being endorsed by The Arthritis Foundation [44] devoted to arthritis as a therapeutic activity for adults living with HOA, to our knowledge, no other comparative study studied its effectiveness on reducing symptoms related to HOA, potentially decreasing their impact in daily life and thus improving quality of life.

The primary objective of this RCT will be to examine the effectiveness of a 12-week knitting program for morning stiffness (primary outcome) and pain relief (secondary outcome) for participants in the knitting in Group 1 compared to participants in the control group assigned to a waiting list (Group 2) in older females (aged 50 to 85 years old) with mild to moderate osteoarthritis of the hands (HOA) after 2 h post-awakening. The secondary objective is to determine if older females with mild to moderate HOA using an adapted knitting program (Group 1) have improved hand and finger strength and physical function, self-efficacy, hand physical activity level and adherence, global patient improvement as well as quality of life (QoL) compared to a waiting list control group (Group 2) over 12 weeks of intervention and after a follow-up at 4 weeks post intervention. Our general hypothesis is that older females with mild to moderate HOA in the 12-week knitting group (Group 1) will have significant improvements in all daily clinical and implementation outcomes compared to the participants in the waiting list (Group 2) after 12 weeks of intervention.

## Methods/design

The following methodology is in full agreement with the SPIRIT (Standard Protocol Items for Randomized Trials) recommendations [45–47] and the Osteoarthritis Research Society International (OARSI) recommendations for RCTs [48] to ensure methodological rigor. Consolidated Standards of Reporting Trials (CONSORT) [49] guidelines will be followed for reporting on the results in a subsequent article.

### Study design

**Fig. 1:**
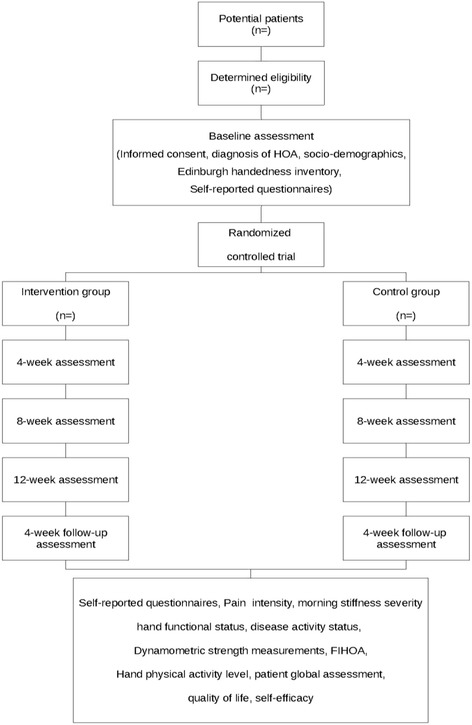
Study design flow chart

The study is a single blind, two-arm RCT with a parallel group design to compare two study groups: 1) a knitting intervention (Group 1) and 2) a waiting list control group (Group 2). The intervention period will be 12 weeks plus a follow-up at 4 weeks post-intervention to measure the retention effect. Since this RCT involves a physical intervention (i.e. knitting program), the therapist, participants, and research coordinator administering the program will not be blinded. A blinded independent assessor will be trained to assess the participants though performance evaluation and self-reported questionnaires given at baseline, 3,6,9,12-week as well as at 4-week follow-up to reduce detection bias. With training and standard operating procedures, it is anticipated that any performance bias due to unblinding will be minimized. The study design flow chart is presented in Fig. 1. The study protocol is approved by the University of Ottawa Research Ethics Board (#H02–16-12).

### Study population

The diagnosis of HOA will be made by a rheumatologist (SZA) and will be consistent with the clinical criteria as set out by the American College of Rheumatology (ACR) classification of HOA [50], the radiologic criteria according to Kallman et al. [51] (1989), and the disease activity criteria according to the Doyle Articular Index [52]. The severity of the HOA will be determined according to ACR classification of HOA and by clinically assessing the presence of (1) pain, aching or joint stiffness, (2) bony enlargement of 2 or more of 10 selected finger joints, (3) bony enlargement of at least a distal interphalageal (DIP) joint, and (4) fewer than 2 swollen metacarpophalageal (MCP) joints, or (5) deformity of at least 1 of 10 selected joints. Presence of OA will be confirmed by a recent X-rays taken of each hand (less than 1 year); films will be interpreted by a radiologist and reports will be sent to the rheumatologist (SZA) to confirm diagnosis and classification.

### Inclusion/exclusion criteria

To be eligible for this RCT, participants will be required to: 1) meet the ACR clinical and radiographic criteria of definite HOA and of mild to moderate severity status [50] and have experienced pain symptoms for at least 3 months, 2) be between 50 and 85 years of age, 3) have a level of morning stiffness of at least 4/10 on a visual analogue scale (VAS) [53] at the time of study entry, 4) display X-ray evidence of joint space narrowing of the hands [51], 5) have no knowledge of knitting (do not know how to knit) or have not knitted in the last 2 months; 6) be available for sessions at the senior’s club twice a week, 7) be able to understand written and verbal English instructions. Participants will be excluded if they: 1) are current active knitters; 2) are unwilling or unable to adhere to the knitting program for 12 consecutive weeks, 3) have other orthopedic, rheumatologic diseases (i.e. inflammatory arthritis, psoriasis arthritis, gout…), or evidence of chondrocalcinosis, 4) had any prior surgery for the finger joints, 5) have any acute disease, such as uncontrolled diabetes mellitus, untreated hypertension, neurological deficits (motor or sensory) or cognitive deficit and mental health conditions, 6) are taking OA medication that is expected to change during the study period, 7) receiving current rehabilitation treatment or any other pain-related treatment besides medication for OA, 8) have received corticosteroid injection of finger joints within the last 6 months, 9) plan to move or relocate within 6 months.

### Recruitment

Three main methods will be used to recruit potential participants. Advertisements in local newspapers will be placed. Information letters will be sent to local rheumatologists and posters will be placed in waiting rooms of each different Ottawa-based rheumatology units and TAS office. Potential participants will be invited to complete an online eligibility/admission questionnaire [53], including sociodemographic, initial intensity, location (e.g. finger joints versus thumb joints; right versus left hand), timing, intensity and duration of morning stiffness and pain [54–56] and handedness information [57, 58] to ensure that they meet the study’s selection criteria prior to randomization. If deemed eligible, participants will be invited to meet the research coordinator to confirm eligibility, sign an informed consent form, and complete baseline evaluations. In a previous RCT on HOA [59], 40 participants over 3 months were recruited, thus the data collection involving a 12-week knitting program for each participant should represent approximately six additional months, leaving 2 months for data analysis and writing the scientific report.

### Group assignment

Eligible participants will be randomly assigned to each group (ratio1:1). Participants of group 2 will have access to the knitting program after the completion of the RCT. Central randomization [60] based on a sequence of computer-generated random numbers (using statistical software SAS macro in SAS 9.3) using a blocking factor (randomly varying between 4 and 6) will be used.

The research coordinator, who is not involved in data collection, will contact the research study Methods Center data manager. Prior to running the randomization program, the data manager will document the participant’s initials (first and last) and date of birth (month and year). After running the program, the data manager will document the intervention assignment with the participant information, assign a study identification (ID) and then inform the research assistant of the assignment and participant ID. This process will help ensure concealment of allocation. After randomization, the participant will be informed of their group assignment.

### Intervention

Participants in both groups will continue with their on-going medical care To measure the true effect of the knitting intervention, active hand exercises need to be avoided since they are proven effective for HOA [7, 9–16, 61]. This may not be possible thus all participants will complete a daily activity log.

Participants in **Group 1** will take part in a low-intensity knitting program, performed as a morning functional activity, which will comprise two components: 1) bi-weekly 20-min knitting sessions at a senior’s club and 2) 20-min home daily knitting sessions for the five remaining weekdays over 12 consecutive weeks (total of 90 sessions). The bi-weekly 20-min knitting session will take place, at **a Senior’s Club in metropolitan Ottawa,** on Tuesday and Thursday mornings with a group of regular members who knit wool blankets for sick children admitted CHEO, a regional Children’s Hospital. The blankets are composed of individual squares that are assembled together. Each wool square takes approximately 20 min for a beginner knitter. Each knitter will be encouraged to knit one wool square per day for a total of seven squares per week. To avoid excessive isometric strength and potential muscle fatigue with the use of small needles that require precise pinching, participants will use specific knitting needle and wool sizes (sizes #6 and #5 respectively according to the Standard Yarn Weight System (**http://www.craftyarncouncil.com/weight.html**). Study participants will learn how to knit with a qualified instructor from Senior’s Club during the knitting sessions which will also be supervised by a trained OT to ensure that the physiological and clinical characteristics of therapeutic exercise are being met. A knitting instructor will ensure that participants follow the prescribed program and if their individual logbooks, where they recorded their daily hand activities and morning stiffness and pain levels, are filled properly. In the event that a session is missed, participants in Group 1 will receive a telephone reminder from the research assistant to encourage them to attend the next session. At the end of 12 weeks, participants will be encouraged to continue on with the program if they gained an improvement of their condition.

Participants assigned to the control group (Group 2) will be placed on a waiting list until the end of the study (12 weeks) plus the 4-week follow-up period. The research coordinator will offer one introductory session to explain how to record any activity in their logbooks as well a weekly telephone follow-up by the coordinator to if participant understood well how to fill them daily. Participants will not be permitted to attend knitting sessions at the senior’s club during the study duration. After the completion of the study duration, the knitting sessions will be available to all study participants. To avoid potential contamination, individuals in Group 2 will have no contact with the individuals registered at Senior’s Club in Group 1. Club membership annual fees ($10/year) will be paid for all study participants in Group 1 and for those in Group 2 only after the completion of the study. Free knitting material and lessons will be provided for all study participants.

### Self-reported and performance-based clinical outcomes outcome measures (Table 1)

**Fig. 2:**
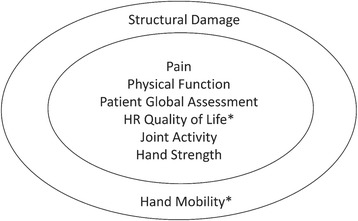
Preliminary set of endorsed core domains for hand osteoarthritis studies. Inner circle: Domains for all settings, i.e., clinical trials of symptom modification, clinical trials of structure modification, and observational studies. Outer circle: Domains for some settings, i.e., clinical trials of structure modification and observational studies. *Domains not mandatory as long as no disease-specific instruments are available. HR: health-related. “Reprinted with permission from The Journal of Rheumatology, Kloppenburg, M., et al J Rheumatol 2015; All rights reserve” ©2015

The clinical outcomes were selected according to the OMERACT framework illustrated in Fig. 2 and include the followings:

**Table 1:** Assessment schedule for primary, and secondary outcome measures

Assessment	Admission	Daily	Baseline	3/6/9 weeks mid intervention	12 weeks end of intervention	4 weeks follow-up
Informed consent (pre-admission)	X					
Diagnosis of HOA based on ACR criteria	X					
Socio-demographics	X					
Edinburgh Handedness Inventory	X					
Self-reported daily morning stiffness intensity (Visual analogue scale: VAS) (primary daily outcome)	X	X	X	X	X	X
Self-reported daily pain intensity (VAS) (intermediate daily outcome)	X	X	X	X	X	X
Pain intensity (Auscan) (periodic secondary measurement)			X	X	X	X
Morning stiffness severity (Auscan) (periodic secondary outcome)			X	X	X	X
Hand functional status/difficulty in activities of daily living (Auscan) (periodic secondary outcome)			X	X	X	X
Disease activity status (periodic secondary outcome)			X	X	X	X
Dynamometric strength measurements (Grip and pinch measurements) (periodic secondary outcome)			X	X	X	X
The Functional Index for Hand Osteoarthritis (FIHOA) (periodic secondary outcome)			X	X	X	X
Hand physical activity level (periodic secondary outcome)		X	X	X	X	X
Patient global assessment (periodic secondary outcome)			X	X	X	X
Quality of life (EQ-5D) (periodic secondary outcome)			X	X	X	X
Self-efficacy (periodic secondary outcome)			X	X	X	X
Physical Activity Enjoyment Scale (PACES) (Group 1 only)				X	X	X
Adherence to knitting program (Daily secondary outcome)			X	X	X	X
7-day Physical Activity Recall (PAR) (Daily secondary outcome)			X	X	X	X

### Self-reported clinical outcomes (Table 1)

**Morning stiffness and pain relief**, using daily visual analogue scale for morning stiffness (primary outcome) and for pain (secondary outcome) will be recorded before (baseline) and after knitting (immediately, 2 h and 4 hours after the 20-min knitting daily morning activity). Daily morning stiffness after 2 h knitting daily morning activity will be our primary outcome measure of this protocol. This choice of primary and intermediate outcomes is based on the feasibility study conducted by the team.

**Functional status**, using Australian/Canadian Osteoarthritis Hand Index (AUSCAN) tool as a validated, reliable and responsive measurement scale for HOA [62–64] was adopted. The AUSCAN includes a 15-item scale, with items grouped into three sub-sections: A) pain intensity, B) stiffness severity and C) hand functional status/difficulty in activities of daily living. It uses a five-point scoring system. A score of 0 represents no pain or no severity in stiffness or no difficulty in performing functional tasks; scores between one and four represent mild to extreme gradations, respectively. Morning stiffness and hand functional status are included in the AUSCAN.

**Hand activity** ***level***, using an adapted 7-Day Physical Activity Readiness (PAR) [65–67] was used. The 7-day PAR is a validated instrument in a calendar format in which a participant can indicate the PA duration (minutes per day) and PA frequency (days per week). An adapted version of the 7-day PAR will be included in the logbooks where physical activity is split into distinct Knitting and other hand activities categories. The logbooks will also include a daily visual analogue scale for morning stiffness and pain intensity before and after knitting, information about hand physical activity level and knitting technique as well as a weekly questionnaire on actual changes in PA, medication intake (not encouraged), habits and adverse events. A similar logbook was created for PA activity other than knitting recording for the participants in the control group 2. ***Patient global assessment*** will be evaluated by asking patients if their condition after knitting program “fully improved”, “partially improved”, “did not improve” or “was worse compared to the beginning of the study” [31]. ***Health-related QoL*** will be assessed using the EuroQoL Index (EQ-5D-5 L) [68] including mobility, self-care, usual activities, pain/discomfort and anxiety/depression subscales.

### Performance-based clinical outcomes (Table 1)

Additionally, the disease activity status will measure the presence of finger inflammation of active joints using a standardized physical assessment.

(https://www.arthritis.ca/healthcare-professionals/standardized-assessment-of-joint-inflammation-(saj) featured by TAS and performed by a therapist from TAS. Two physical assessments will also be performed: 1) The Functional Index for Hand Osteoarthritis (FIHOA) [69] will also be used as a performance-based physical function measure as well as 2) dynamometric strength measurements for hand/fingers [40].

### Implementation outcomes

The following implementation outcomes are not represented in the OMERACT framework (fig. 2). ***Self-efficacy*** will be assessed using The Stanford Arthritis Self-Efficacy Scale (ASES) [33], a ten-point scale ranging from one (very uncertain) to 10 (very certain). Level of ***knitting enjoyment*** will be measured using Physical Activity Enjoyment Scale (PACES) for participant in group 1 only [70]. ***Adherence to PA/knitting program*** will be estimated as the number of knitting sessions attended at the Club and performed at home divided by the number of knitting sessions prescribed (84 sessions), as recorded in the participants’ logbooks, using the 7-Day PAR calendar [65] as well as attendance at the senior’s club. The number of wool squares that were knitted will be also reported in the participants’ logbooks. However, knitting technique will be recorded how the participants hold the stabilizing and mobile needle and which hand is used by the trained OT. Adverse events observed will be also compiled. All these clinical and implementation outcomes measures were pilot tested [43].

Please insert Table 1 Assessment schedule for primary, and secondary outcome measures.

### Sample size calculation

The most relevant information on the standard deviation available was from the feasibility study. Even though the sample of this feasibility was small (*n* = 5), the participants and the environment in which the study was conducted and the measurements taken are directly in alignment with that for the proposed RCT. Each patient was assessed at 2 h. The reported Area Under the Curve (AUC) score, as a proportion of the 42 day potential AUC score of 420 (42 days with maximum score of 10; 10 cm Visual Analogue Scale (VAS) [53], at 2 h post had a mean of 0.188 and standard deviation of 0.261 cm, based on the observed AUC scores (i.e. mean and standard deviation of the 5 AUC scores of the participants were calculated). The large effect size 0f 0.8 was determined based on discussions among the study investigators and other experts in the field. A large effect size was believed to be needed since a substantive morning stiffness improvement would be required to lead to potentially important improvements in subsequent daily functioning; whereas smaller changes in effect size were not believed to be substantive enough to impact these activities of daily functioning. To detect an effect size of 0.8, with power of 80% and level of significance of 0.05, a sample size of 28 participants per group will be required. No dropouts in the pilot were experienced and none are expected in the main study given the short duration of the study; however a 5% dropout is assumed and a sample size of 30 per group will be recruited.

No dropouts in the feasibility study occurred and none are expected in the main study given the short duration of the study; however a 5% dropout is assumed and a sample size of 30 per group will be recruited.

### Measurement frequency

Clinical and implementation evaluations will be performed by the independent and trained research therapist at baseline, 3 weeks, 6 weeks, 9 weeks, and end of study (12 weeks) + 4 weeks FU for participants in both groups. Measurement instruments will be calibrated every month before the evaluation sessions and will be administered in a randomized sequence. Independent evaluation sheets will be used for each patient at each assessment session in order to minimize recording bias. All measurements will be performed through the use of electronic self-reported questionnaires on a laptop using the survey website. The evaluations will take approximately 60 min to complete and will be performed in a private assessment room located at University of Ottawa to optimize blinding of the evaluator.

### Statistical analysis

Descriptive statistics will be used to summarize the study variables and to assess the distributional assumptions of the statistical techniques used. An intention to treat analysis will be conducted for all the data analyses.

A per protocol analysis will be considered as part of the sensitivity analyses. The primary outcome is the area under the curve (AUC) at 2 h for stiffness. That is, stiffness at 2 h post-awakening from sleep will be assessed each day for 12 weeks, and the AUC over this 12 week period will then be calculated. Similar AUCs will be calculated for stiffness at 0 h (at awakening), 4 h (post-awakening) and evening. Also, similar AUCs for the secondary outcomes pain and function will be calculated.

For the primary outcome (AUC at 2 h for stiffness) participants in the knitting group will be compared to participants in the control group using independent Student’s t-test with the pooled or separate variance estimate as appropriate. For the primary and secondary outcomes, the area under the curve (AUC) for stiffness, pain and function will be compared using a 2-way analysis of variance (ANOVA) with the between factor group (knitting vs control) and within factor time (0 h, 2 h, 4 h, evening). Tukey’s [71] honest significance difference (HSD) will be used to make specific pairwise comparisons. The secondary outcomes, AUSCAN overall, AUSCAN pain, AUSCAN stiffness, AUSCAN function, hand and fingers strength and physical function, self-efficacy, hand physical activity level and adherence, global patient improvement as well as QoL will be compared using a 2-way analysis of variance (ANOVA) with the between factor group (knitting vs control) and within factor time (baseline, 3 wk., 6 wk., 9 wk., 12 wk. and 4-wk Follow-Up). Again, Tukey’s HSD [71] will be used to make specific pairwise comparisons. As a secondary analysis, additional time assessments to 16 weeks will be included in the above analyses to assess follow-up. An alpha significance of 0.05 was chosen for all analyses. Mixed models repeated measured will be used to accommodate missing data. No interim efficacy or subgroup analyses are planned.

## Discussion

### Strengths and limitations

The proposed study is a rigorous single blind, two-arm RCT with a parallel group design to assess the efficacy of a knitting program, which is a low-cost, community-based, innovative and accessible intervention at reducing hand impairment, improving occupational performance, as well as enhancing the self-efficacy, coping strategies and QoL of the older women with HOA [5,6]. The proposed knitting program is based on: 1) evidence stemming from physiological studies [34–36]; 2) recommendations for individuals over 50 years old with OA [37]) and specifically with HOA [12]; 3) evidence-based general exercise protocols for HOA [5, 38, 39] and OA of the thumb [40–42]. The movements typically involved in hand therapy for HOA are also involved in knitting. The previous case study [43] and feasibility study conducted by co-investigators of this protocol suggest that knitting represents a promising, functional and meaningful activity for elderly women suffering from HOA to decrease morning stiffness and pain via its effect on biological, psychological and social factors [22]. The meaning attributed to this activity may contribute to increase adherence to the “exercise program”. Optimal therapeutic and functional exercises dosage is difficult to select, especially considering that a study on knitting for HOA has not been conducted as of yet [7]. A review of existing protocol [5, 38, 39], and previous RCTs on therapeutic exercises [9, 14] were considered to inform the selection of the proposed knitting program intensity, frequency, duration. However, the existing protocols need to be adapted so they are tailored with to the therapeutic goals and knitting specificity. However, the knitting program was considered to be performed daily (not 2 to 3 days a week) since the immediate symptom effect lasts for four consecutive hours. Similarly to acetaminophen, knitting could be used as a temporarily pain or morning stiffness relief minus potential side effects of the drug often associated in adults [72]. However, it is hypothesized that regularity of any type of land-based exercise program seems more important than intensity to improve joint health [43]. A total study duration of 16 weeks (12-week knitting program plus a follow-up period of 4 weeks) is justified since it is not ethical to put a study participant on a long-term waiting list without an active intervention. The timeframe of the intervention and adherence in the long term beyond the 4-week follow-up won’t be assessed. This compromise is mandatory in order to use a waitlist as control condition.

To minimize the potential misclassification bias, a rheumatologist involved as a co-investigator and specialized in HOA will make sure each participant enrolled in this RCT has a definite diagnostic of HOA. The ACR classification [50], the radiologic criteria according to [51], and the disease activity criteria according to the Doyle Articular Index [52] will be taken into consideration.

Assessments will include a range of clinical as well as implementation outcome measures. However, there is an increased risk of Type-1 error due to the presence of multiple outcomes. According to the adapted OMERACT group [73], all HOA studies should assess morning stiffness, pain, physical function, patient global assessment, joint activity and hand strength. In a recent systematic review [74], the AUSCAN, FIHOA, VAS pain, grip and pinch strength, and pain on palpation were the outcome measures most frequently used for HOA and provided supporting evidence for good metric properties [75–77].

Potential information bias includes the imprecision of self-reporting outcomes [78]. The inclusion of performance-based physical function measures (e.g. Functional Index for Hand OA (FIHOA) [69] rather than solely measuring self-reported measures (e.g. AUSCAN function subscale) [62, 63] would help address these limitations. The primary and secondary outcomes, respectively morning stiffness and pain, are self-reported, but represent important unwanted symptoms that have functional consequences that affect the QOL of individuals with HOA. Objective measures of these particular symptoms are difficult to obtain. However, pain and morning intensity using a visual analogue scale is recognized as a gold standard, since the nature of pain is a subjective perception/experience. Unfortunately, no technology can measure this personnel sensation outcome [79]. In addition to self-reported VAS measurements of pain and morning stiffness intensity before and after knitting, the number of squares knitted will be recorded in logs books to capture the knitting intensity/quantity. Daily self-reported VAS measurements of pain and morning stiffness intensity before and after knitting are important to be recorded in the participant’s logbook, because these symptoms have a negative impact in their functional activities and QoL. Logbooks is a tool that can be useful to minimize potential recall biases. It might also be difficult to discriminate between pain from fingers versus from thumb for each hand assessment. This is why it important to take into consideration intensity, location, timing and duration of pain and joint stiffness [55] in the assessment in each finger affected by HOA.

This proposed RCT is necessary to address questions of clinical and scientific importance for rehabilitation specialists in improving the QoL of elderly women with HOA living in Canada. The results of this study will likely be generalizable to older women with mild to moderate HOA. If the results of this proposed RCT are positive, this accessible activity may be generalized to individual home-based knitting or group-based knitting in existing social clubs. The results of this RCT may give evidence to health professionals of an effective alternative physical activity to suggest for their patients with HOA.

## Conclusion

This knitting RCT has substantial potential to enhance our understanding of this functional activity on the management of HOA, refine dosage and adherence recommendations in older women for this prevalent disease and to ultimately reduce the burden of disability in this aging population. This proposed RCT will contribute to the knowledge of the effect of knitting for individuals with HOA on self-reported and performance-based, HOA symptoms, level of QoL as well as fingers strength hand function and their self-efficacy and exercise adherence.

This proposed knitting RCT could also determine if this functional self-management activity has a daily and punctual effect on symptoms (i.e. HOA morning stiffness or pain) relief only (similarly to acetaminophen a pain reliever) or has also an effect on clinical and implementation outcomes,

This study could also make knitting the standard-of-community-based-care for older women with HOA. Moreover, other rehabilitation or functional interventions could be examined for older people with other chronic diseases.

## Abbreviations

ACR: American College of Rheumatology
ASES: Arthritis Self-Efficacy Scale
CHEO: Children Hospital of Eastern Ontario
CONSORT: Consolidated Standards of Reporting Trials
DIP: distal interphalageal
FIHOA: Functional Index for Hand Osteoarthritis
HOA: Hand Osteoarthritis
HR: health-related
HSD: honest significance difference
ID: identification number
MCP: metacarpophalageal
OA: Ostoarthritis
OT: occupational therapist
PA: physical activity
PAR: Physical Activity Readiness
QoL: quality of life
RCT: randomized controlled trial
SPIRIT: Standard Protocol Items for Randomized Trials
VAS: visual analogue scale
